# Outcomes Reported After Surgery for Cauda Equina Syndrome: A Systematic Literature Review

**DOI:** 10.1097/BRS.0000000000002605

**Published:** 2018-08-15

**Authors:** Nisaharan Srikandarajah, Martin Wilby, Simon Clark, Adam Noble, Paula Williamson, Tony Marson

**Affiliations:** ∗ Institute of Translational Medicine, University of Liverpool, Liverpool, United Kingdom; † Department of Neurosurgery, Walton Center NHS Foundation Trust, Liverpool, United Kingdom; ‡ Institute of Psychology Health and Society, University of Liverpool, Liverpool, United Kingdom.

**Keywords:** cauda equina syndrome, core outcome set, neurology, neurosurgery, orthopedics, outcome domains, outcomes, Prisma, spine surgery, surgery, systematic literature review

## Abstract

**Study Design.:**

This is a systematic literature review following the Preferred Reporting Items for Systematic Reviews and Meta-analyses guidelines.

**Objective.:**

To assess the outcomes reported in trials and observational studies of surgery for cauda equina syndrome (CES), and to inform the development of a core outcome set.

**Summary of Background Data.:**

Scoping searches revealed that there were inconsistencies in which outcomes were reported and how they were measured in research studies for patients who had undergone surgery for CES.

**Methods.:**

Ovid Medline, Embase, CINAHL Plus, and trial registries were searched from January 1, 1990 to September 30, 2016 with the term “cauda equina syndrome.” Inclusion and exclusion criteria were applied according to study design, diagnosis, procedure, publication date, language, and patient age. Data extracted included demographics, study design, the outcomes reported, and their definition. We also assessed variation in the use of terminology for each outcome domain.

**Results.:**

A total of 1873 articles were identified, of which 61 met the inclusion criteria. Of these, 737 outcomes reported verbatim were categorized into 20 core outcome domains and 12 subdomains with a range of 1 to 141 outcomes per outcome domain or subdomain. The most commonly reported outcomes were bladder function (70.5%), motor function (63.9%), and sensation (50.8%). Significant variation in the terms used for each outcome was documented, for example, bladder function outcome domain had 141 different terms.

**Conclusion.:**

There is significant heterogeneity in outcomes reported for studies after surgery for CES patients. This indicates a clear need for the development of a core outcome set, which has been registered as number 824 on the COMET (Core Outcome Measure in Effectiveness Trials) database.

**Level of Evidence:** 1

## Abstract 2

Supplemental Digital Content is available in the text

Cauda equina syndrome (CES) is mainly caused by compression of the lumbosacral nerve roots below the conus medullaris. Clinically, symptoms and signs include low-back pain, saddle anesthesia, unilateral or bilateral sciatica, and motor weakness of the lower extremities with bladder and bowel dysfunction.^1,2^ However, CES is a clinical-radiological diagnosis as clinical signs are not particularly sensitive to a CES diagnosis.^3,4^ A lumbo-sacral magnetic resonance imaging (MRI) is required for diagnosis. Gleave and McFarlane^5^ stressed the importance of categorizing CES into CES incomplete (CESI) and CES complete with urinary retention (CESR) (Figure 1). It is deemed a surgical emergency and there have been numerous publications and debates relating to the ideal timing for surgery.^6–9^ It can result in permanent damage to nerve roots resulting in long lasting or permanent disabling symptoms.^2^

**Figure 1:**
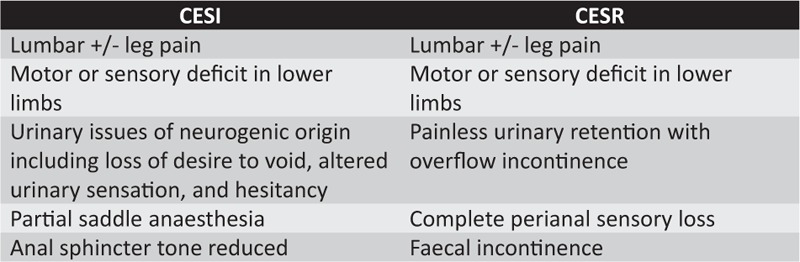
Symptoms relating to CESI and CESR.

There is no consultation with patients in the literature regarding importance of outcomes for CES. In addition, there is little known about the long-term outcomes, which was highlighted by Korse et al,^10^ who independently decided to focus on outcomes of micturition, defecation, and sexual function. Bias in studies, lack of universal definitions, and incomplete follow-up data were seen in this systematic review.

The problems with not having a core outcome set (COS) includes:

(i) Patients are not included so important outcomes to them may not be measured. This has been witnessed in other healthcare areas such as childhood asthma and esophageal cancer.^11–14^
(ii) Lack of a consistent approach makes individual studies difficult to interpret and put into context of other studies.
(iii) Possibility for synthesizing evidence in a systematic review and meta-analysis are diminished ^15^.
(iv) Waste and inefficiency. It is reported that 85% of research funding is wasted across the research cycle with key sources related to outcomes; important outcomes are not assessed, published research fails to set its position when compared with all previous similar research and 50% of planned study outcomes are not reported.^16^

At present, there is no COS for CES, which is to the detriment of patients and health services. The aim of this systematic literature review is to inform the future development of a COS by identifying all documented outcomes for patients after surgery in CES, identify if they are defined, and to assess what variability there is. The systematic literature review is the first step to inform the development of a COS^14^ for patients who have undergone surgery for CES to be used in research and in practice.

## METHODS

This study has been registered as 824 on the COMET (Core Outcome Measures in Effectiveness Trials) website (http://www.comet-initiative.org/studies/details/824). Table 1 lists the inclusion criteria applied to the search strategy.

**TABLE 1:** Inclusion Criteria for the Systematic Literature Review

Diagnosis of CES
Patients have undergone surgery for the pathology causing CES
Randomized controlled trials, nonrandomized controlled trials, prospective and retrospective cohort studies, and case series
Human studies
English language
Five or more patients
Published between 1990 to September 30, 2016
Adult patients aged 16 years and above

CES indicates cauda equina syndrome.

### Search Strategy

We searched Medline, Embase, and CINAHL Plus (Cumulative Index to Nursing and Allied Health Literature). The search strategy for each database is available in Appendix 1. Online trial registries included Clinical Trials.gov, EU clinical trials registry and the ISRCTN (*International Standard Randomized Controlled Trials Number)* registry. The trial registries were searched for any completed or ongoing trials in surgery for CES and no relevant studies were found. Only case reports and abstracts were excluded in the initial search term as we wanted studies with five or more patients. We only included studies published after January 1, 1990 to keep investigation (post-MRI era) and surgical management of CES in line with current medical practice. Citations were collated with Endnote X7 referencing program (Thomson Reuters, New York, NY) and duplicates removed.

### Data Extraction

Titles and abstract were initially screened by NS to identify potential studies for inclusion, for which full text articles were obtained for further assessment. Approximately, 10% of included articles were randomly checked for suitability by clinical supervisors and any discussion regarding uncertainty of eligibility criteria applied to the search results was discussed with them (SC, MW, and TM). A data extraction form was used to collect data on study design and location, patient demographics, timing of operation, definition of CES, diagnosis, etiology, surgical procedure, follow-up duration, outcome terminology, outcome definition, and assessment tool.

### Terminology

Below are the definitions for the main terms used in the analysis of this systematic literature review.

1. Core outcome domain- The overall category to which similar subdomains and outcomes are listed under. The outcome domains that we have used in this article have been linked to the high level set of outcome categories used for annotation of Cochrane reviews^17^ (http://linkeddata.cochrane.org/linked-data-project/metadata-and-vocabularies/outcomes) and through discussion with the COMET initiative team. These are listed in bold in Table 3.
2. Subdomain- A subcategory of a Core outcome domain to which similar outcomes are listed under. These are listed in normal script in Table 3.
3. Outcome- An outcome documented in an article after a patient has had an operation for CES. For example, nervous system (core outcome domain)> bladder function (subdomain)> urinary incontinence (outcome).
4. Variations- Variations were also documented, which means the number of different terms used to define a core outcome domain or subdomain. An example of a variation is given in the superscript of Table 4.
5. Outcome definition- this was categorized as “no definition” or “definition present.” If a definition was present it could be subjectively a complete or partial definition but was recorded as “definition present.” “No definition” indicates the outcome domain was mentioned with no accompanying definition in the article or assessment tool. An example of how outcome definition was done is given in the superscript of Table 4.

**TABLE 3:** Core Outcome Domains (in Bold) and Subdomains

**Mortality**	**Role Functioning**
**General Disorders**	**Social functioning**
**Nervous System Outcomes**	**Emotional functioning**
Bladder Function	**Global quality of life**
Motor Function	**Hospital use**
Sensation	**Need for intervention**
General Neurology	**Adverse events**
Lower Back Pain	**Infection**
Leg Pain	**Skin and subcutaneous tissue**
Bowel Function	**Vascular**
Perianal sensation	**Outcomes related to neoplasms**
Perianal Tone	**Urological and renal**
Reflexes	**Cardiac**
**Physical Functioning**	**Blood and lymphatic**
Sexual Function	**Respiratory**
Walking	**Gastrointestinal**

**TABLE 4:** Raw Data for Each Outcome Showing How Many Studies Each Outcome is Reported in, the Total Number of Outcomes, the Variations for Each Outcome, if a Definition is Present in the Reported Studies and the Number of Assessment Tools for the Reported Outcomes. Outcomes are Listed in Order of Decreasing Frequency of Reported Studies

Outcome Domain	Reported 61 Studies, N (%)	Total Number of Outcomes	Number of Variations	Definition Present in Reported Studies (%)	Assessment Tool in Reported Studies (%)
Bladder function (nervous system)	43 (70.5)	141	87^*^	25 (58.1)^†^	13 (30.2)
Motor function (nervous system)	39 (63.9)	62	36	9 (23.1)	16 (41)
Sensation (nervous system)	31 (50.8)	53	26	6 (19.4)	6 (19.4)
Bowel function (nervous system)	28 (45.9)	60	47	7 (25)	8 (28.6)
Leg Pain (nervous system)	27 (44.3)	32	16	5 (18.5)	7 (25.9)
Lower-back pain (nervous system)	26 (42.6)	31	13	4 (15.4)	9 (34.6)
General neurology (nervous system)	22 (36.1)	31	21	3 (13.6)	8 (36.4)
Skin and subcutaneous tissue	19 (31.1)	22	15	5 (26.3)	0 (0)
general disorders	19 (31.1)	44	36	6 (31.6)	6 (31.6)
mortality	18 (29.5)	25	13	6 (33.3)	0 (0)
Perianal sensation (nervous system)	17 (27.9)	23	16	5 (29.4)	0 (0)
Sexual function (physical functioning)	16 (26.2)	46	41	6 (37.5)	6 (37.5)
Walking (physical functioning)	16 (26.2)	28	25	3 (18.8)	5 (31.3)
Adverse events	12 (19.7)	16	12	8 (66.7)	0 (0)
Role functioning	11 (18)	20	20	3 (27.3)	7 (63.6)
Perianal tone (nervous system)	11 (18)	16	13	2 (18.2)	0 (0)
Need for intervention	10 (16.4)	13	13	6 (60)	0 (0)
Infection	10 (16.4)	11	8	1 (10)	0 (0)
Vascular	8 (13.1)	13	5	0 (0)	0 (0)
Hospital use	5 (8.2)	8	6	0 (0)	0 (0)
Global quality of life	5 (8.2)	8	6	3 (60)	4 (80)
Reflexes (nervous system)	4 (6.6)	7	7	0 (0)	0 (0)
Emotional functioning	4 (6.6)	7	7	1 (25)	3 (75)
Respiratory	4 (6.6)	4	5	0 (0)	0 (0)
Outcomes relating to neoplasms	3 (4.9)	5	3	0 (0)	0 (0)
Urological and renal	3 (4.9)	3	3	0 (0)	0 (0)
Cardiac	3 (4.9)	3	2	0 (0)	0 (0)
Social functioning	2 (3.3)	2	2	0 (0)	2 (100)
Blood and lymphatic	2 (3.3)	2	2	0 (0)	0 (0)
Gastrointestinal	1 (1.6)	1	1	0 (0)	0 (0)

^*^An example of analyzing the variation of terminology used for bladder function outcome domain: “urinary incontinence” “bladder dysfunction” and “urinary retention” are 3 variations of the way this outcome domain is described.

^†^Two examples of how bladder function outcome domain was classified with definition present: (i) retention of urine *–* “the inability to pass urine necessitating urinary catheterization.” This study was retrospective and relied upon adequate documentation in the patients’ clinical notes. Residual urine volumes were only available in 11 patients (all greater than 300 mm) whereas 24 patients were documented to be in urinary retention. Urinary retention at follow-up comprised those patients requiring catheterization to enable them to empty their bladder and also those patients who reported incomplete bladder emptying (McCarthy et al,^49^). (ii) Urine retention diagnosis was clinical (a bladder that required catheterization). (Foruria et al^35^)

## RESULTS

A total of 1873 articles were identified by electronic database searches.

1. Medline (650)
2. Embase (949)
3. CINAHL Plus (239)
4. Registries (35) included Clinical Trials.gov (5), EU clinical trials registry (12) and ISRCTN (*International Standard Randomized Controlled Trials Number)* registry (18).

**Figure 2:**
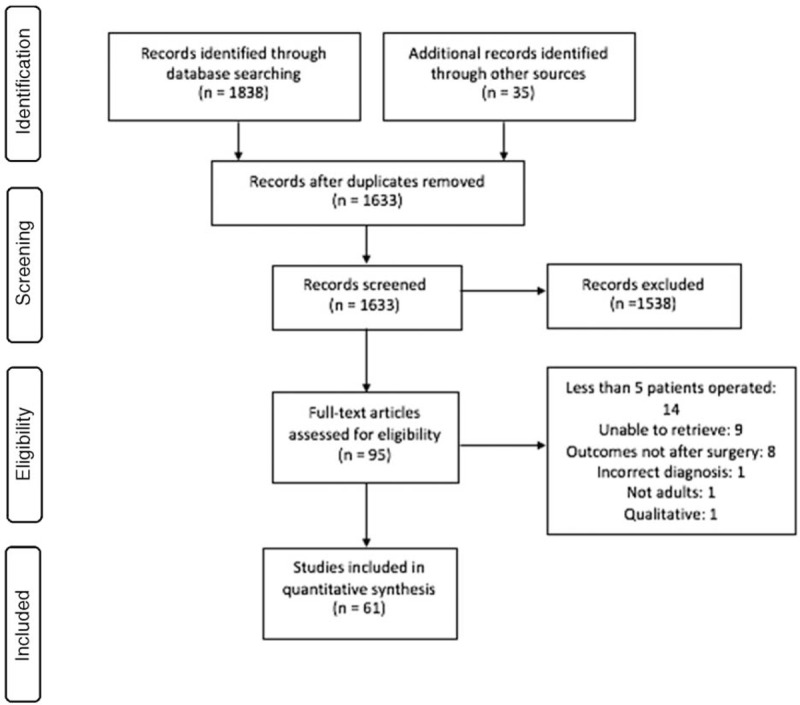
PRISMA flowchart for online databases.

The Preferred Reporting Items for Systematic Reviews and Meta Analyses (PRISMA) flowchart in Figure 2 shows the process during the systematic literature review. Following inclusion criteria in Table 1 resulted in 1838 articles plus the 35 studies from the online registry search giving a total of 1873 studies. Moreover, 10% of included studies were reviewed by a supervisor (MW and SC) to assess if inclusion criteria had been applied adequately and agreement was achieved after discussion amongst us. Uncertainty regarding eligibility of certain full text articles for inclusion were discussed with the clinical supervisory team (MW, SC, and TM) and settled leading to 61 included articles. Thirty-four articles were excluded after the full text was obtained and the reasons for this were given as in Figure 2.

Summary details, patient demographics, and how many studies they were reported in out of the 61 included studies are detailed in Table 2. Most studies (90.2%) were retrospective. CES was not defined in 20 studies (32.8%). Even in the articles where CES is defined there were many differing definitions. The most common definition was CESI and CESR as described in Figure 1.

**TABLE 2:** Summary Characteristics and Demographics of Included Studies

Characteristic (Number of Studies Reported)	Value
Study design (61)	
Retrospective cohort	55
Prospective cohort	6
Location (61)	
Europe	32
North America	15
South America	1
Asia	13
Single center	57
Year of publication (61)	
1990–1995	5
1996–2000	4
2001–2005	10
2006–2010	16
2011–2016	26
Mean follow-up period postsurgery (54)	8.4 yrs
Range	1–38 yrs
Median number of CES patients (61)	14
Range	5 to 11,207
Mean age (53)	45.5
Range	20.5–70
Median follow up (43)	31 months
Range	postoperative–29 yrs
CES definition (61)	
Defined	41
Not defined	20
Diagnostic main investigation (54)	
MRI	44
CT	9
Myelogram	1
Etiology (59)	
Disc herniation	34
Degenerative	4
Postoperative complication	3
Trauma	7
Tumor	6
Other	2
Main surgical method (51)	
Laminectomy and discectomy	15
Laminectomy	14
Laminectomy and instrumentation	12
Microdiscectomy	8
Other	2

CES indicates cauda equina syndrome; CT, computed tomography; MRI, magnetic resonance imaging.

A total of 737 outcomes were reported in the 61 included articles.^9,18–78^ For ease of analysis in this study, these reported outcomes have been categorized to one of the 20 core outcome domains (Table 3). The nervous system core outcome domain had 10 subdomains, and the physical functioning has two subdomains (Table 3). The number of different variations in the description of outcomes can be seen in Table 4 linked to the outcome domains.

**Figure 3:**
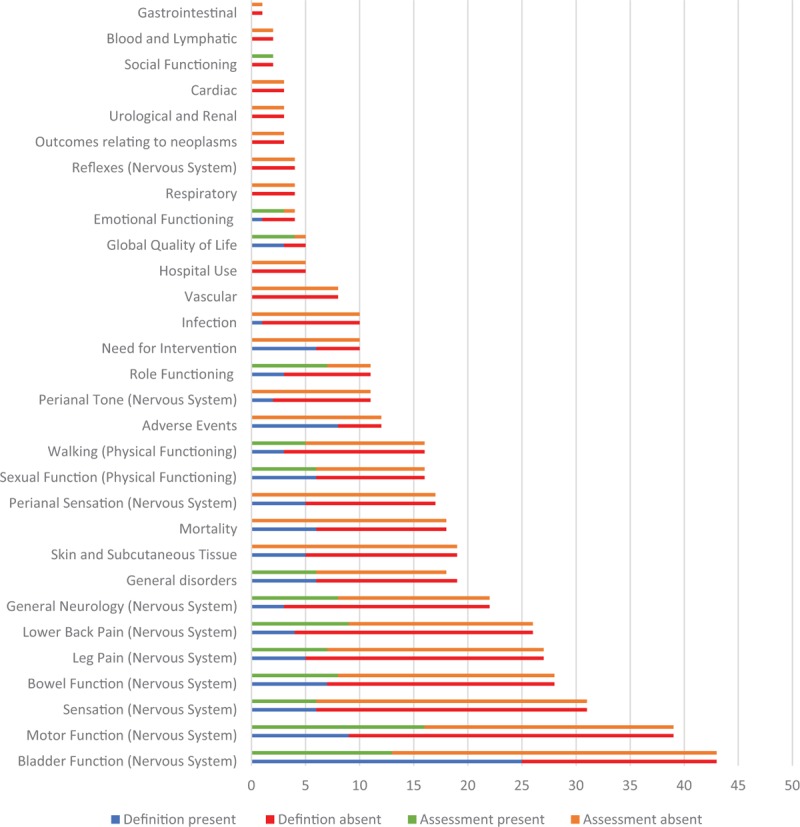
Stacked bar chart showing total number of articles where the outcome was reported and the proportion of those defined (blue) and those not defined (red). Moreover for each outcome the number of articles which have used an assessment tool for a reported outcome (green) and the number that have not (orange). Outcomes are listed from most to least reported.

Figure 3 shows the number of articles in which specific outcomes were reported. Bladder function, motor function, sensation, bowel function, leg pain, and lower-back pain were the most commonly reported in descending order. They are all within the nervous system core outcome domain. Moreover, for each outcome, the number of articles where it is defined and not defined is documented. Figure 3 also shows the number of articles where the reported outcome had an assessment tool or not.

Table 4 shows the raw data for each outcome showing how many studies each outcome is reported in, the total number of outcomes, the number of variations in the description of the outcome if a definition is present or not in the reported studies, and the number of assessment tools for the reported outcome. Table 5 shows the various assessment tools used for each outcome.

**TABLE 5:** Assessment Tools are Listed in Alphabetical Order for the Corresponding Reported Outcomes

Outcome Domain	Assessment Tools
Bladder function (nervous system)	25-Item questionnaire^36^/ Bristol female lower urinary tract/ Cystometry/ Functional Independence Measurement/ Gibbon's criteria/ Gleave and McFarland, 1990/ Hannover pelvic scoring system/ International Continence Society male questionnaire/ Japanese Orthopedic Association score / Modified Odom's criteria/ Short-form Incontinence Questionnaire/ Urodynamics
Motor function (nervous system)	American Spinal Injury Association Score/ Frankel grading/ Gibbon's criteria / McCormick scale/ MRC grading/ Modified Odom's criteria
Sensation (nervous system)	American Spinal Injury Association Score/ Frankel grading/ Gibbon's criteria / McCormick scale/ Modified Odom's criteria/ Nanko evaluation system
Bowel function (nervous system)	25-Item questionnaire^36^/ Chronic idiopathic constipation index / Fecal incontinence questionnaire (Jorge et al 1993)/ Functional Independence Measurement/ Hannover pelvic scoring system/ Modified Odom's criteria/ Nanko evaluation system/ Short-form Incontinence Questionnaire
Leg pain (nervous system)	Benoist et al 1993/ Japanese Orthopedic Association score/ Visual Assessment Score
Lower back pain (nervous system)	Low Back Outcome Score/ Oswestry Disability Index/ Short-form Health Survey 36/ Visual Assessment Score
General neurology (nervous system)	American Spinal Injury Association Score/ Baba et al, 1995 study questionnaire/ Frankel grading/ Gibbon's criteria/ Japanese Orthopedic Association score/ McCormick's scale
General disorders	Epstein & Hood/ Nanko evaluation system/ Prolo economic and functional scale/ Short Form Health Survey 36/ Spengler classification/ Visual Assessment Score
Sexual function (physical functioning)	International index of erectile function/ Male sexual health inventory/ McCormick scale/ Modified Odom's criteria/ Nogueira et al 1990/ Sheffield Female pelvic floor questionnaire/ Japanese Orthopedic Association score
Walking (physical functioning)	Baba et al 1995/ Functional Independence Measurement/ Japanese Orthopedic Association score/ McCormick scale/ Short-form Health Survey 36
Role functioning	Chronic idiopathic constipation index/ Kirkaldy Willis classification/ Nanko evaluation system/ Oswestry Disability Index/ Prolo economic and functional scale/ Short-form Incontinence Questionnaire
Global quality of life	25-Item questionnaire^36^/ Oswestry Disability Index/ Short Form Health Survey 36
Emotional functioning	Functional Independence Measurement/ Kelleher et al 1997 questionnaire/ Short-form Health Survey 36
Social functioning	Kelleher et al 1997 questionnaire/ Short-form Health Survey 36

## DISCUSSION

This systematic review shows that there is significant heterogeneity in the outcomes measured for patients who have undergone surgery for CES with no consensus.

Most of the evidence regarding outcomes for CES patients after surgery is derived from level 4 evidence, namely, single centre retrospective cohort review studies. The average data collection period was over 8 years with a median number of 14 patients per study, which highlights the rare nature of the condition and difficulty in collecting meaningful data retrospectively. This feeling is also echoed by Todd and Dickson, 2016.^79^ Since 1990, the number of publications analyzing outcomes after an operation for CES have increased with the most being produced in the last 5-year period (43.5%). Median follow up was at 31 months reflecting the deficiency in the literature for any long-term outcomes.

The main investigation is MRI, which reflects the systematic literature review focusing on studies from 1990 onwards. Before this there may have been a reliance on myelography and CT to radiologically identify CES compression. The main etiology is disc herniation. There are no studies in the literature documenting the exact distribution of CES aetiology but the most common cause is believed to be because of disc herniation.

Poor definition of CES has been previously highlighted in a systematic review of the literature.^80^ Twenty studies (32.8%) did not define this and of the 41 studies where a definition was present, there was significant heterogeneity in the definitions. The most common definition for CES in this review was CESI and CESR.^5^ If a study fails to define CES then we are unsure of the condition to which the outcomes of the study belong to.

Most common surgical method in studies was a laminectomy and discectomy as seen in Table 2 but there were other studies that predominantly performed surgery *via* a microdiscectomy. Laminectomy alone, or with instrumentation was also mentioned for CES patients. In fact, now there is an increase in the popularity of endoscopic lumbar discectomy procedure ^45^ that adds to the range of procedures available when dealing with CES secondary to disc herniation. There is no consensus in the literature as to a specific decompressive procedure to be used for CES secondary to compressive pathology. This is also another factor that may affect outcomes for these patients.

In total, there were 737 outcomes reported verbatim and categorized into 20 core outcome domains and 12 subdomains. Instead of the same term being used for each outcome, there exists 507 variations in terminology (Table 4). In addition, most of the outcomes in the included articles have no definition. Except bladder function, adverse events, need for intervention, and global quality of life, all other outcomes had “no definition” in the majority of the included articles (Figure 3). This highlights that there is significant heterogeneity in not only the outcome terminology used but the level to which it is defined in the literature. Except global quality of life, emotional functioning, role functioning, and social functioning, most outcomes did not have an assessment tool in most of the articles (Figure 3). Fourteen of the outcome domains/subdomains we categorized had multiple different assessment tools used for each of them as seen in Table 5. There is a lack of uniformity over which assessment tool is best suited for each outcome in the literature. If outcomes are being measured with different scales, scoring systems, and questionnaires then it would be difficult to synthesize these results for meaningful analyses.

There is significant heterogeneity of the outcomes for patients who have undergone an operation for CES, how they are defined and measured in the literature. Bladder function, motor function, sensation, bowel function, leg pain and lower-back pain outcomes are the most reported. They are all physiological core domains, which have been prioritized in the literature over the other core domains that relate to life impact, mortality, resource use, and adverse events. However, there has not been consultation with key stakeholders regarding what outcomes are the most important to be justifying this practice. Involvement of key stakeholders through an iterative process has been employed in Rheumatology through OMERACT (Outcome MEasures in Rheumatology) and in Women's Health through the CROWN (CoRe Outcomes in Women's and Newborns health) initiative ^81,82^ (http://www.omeract.org/; http://www.crown-initiative.org). They have come a long way from developing COS to achieving a level of homogeneity among similar studies to increase the quality and yield of their research. This needs to be achieved for patients who have undergone surgery for CES.

## LIMITATIONS

The systematic literature review was carried out by the main author (NS). Uncertainties and discrepancies were discussed with the research team (PRW, TM, MW, SC, and AN). Only English language articles were included. It would have been beneficial to have another independent group conduct the search strategy and data extract independently and to compare the results achieved. Because of the limitation of resources this was not performed.

## CONCLUSION

There is significant heterogeneity in outcomes reported for studies after surgery for CES patients and the methods by which they are measured. This indicates a clear need for the development of a COS and the results of this systematic literature will be combined with the results of outcomes sourced from CES patients in qualitative interviews. All outcomes will then be prioritized through a Delphi process and consensus meeting to develop a core list of outcomes determined to be of most importance by key stakeholders.

**Key Points**

- For patients who have had an operation for CES there are inconsistencies in the outcomes reported, defined, and assessed between studies.
- Because of the heterogeneity of outcomes reported, defined, and assessed we are unable to synthesize the results for a meta-analysis.
- The outcomes have not been validated in the literature by key stakeholders as being important to them.

## Supplementary Material

Supplemental Digital Content
