# Supplementary material for: Outcomes Reported After Surgery for Cauda Equina Syndrome: A Systematic Literature Review
Source: Spine (Phila Pa 1976). 2018 Aug 15;43(17):E1005–13. doi: 10.1097/BRS.0000000000002605 (PMC6104724; doi:10.1097/BRS.0000000000002605)
Supplement: Supplemental Digital Content [file brs-43-e1005-s001.docx]

**Appendix 1**: Database Search Strategy.

Ovid Medline 30/9/16

| **Search #** | **Search term** | **Results** |
| --- | --- | --- |
| 1 | exp Polyradiculopathy/ | 2485 |
| 2 | (case report or abstract).mp. [mp=title, abstract, original title, name of substance word, subject heading word, keyword heading word, protocol supplementary concept word, rare disease supplementary concept word, unique identifier, synonyms] | 1980773 |
| 3 | Animals/ | 6104266 |
| 4 | 1 not 2 | 2119 |
| 5 | 4 not 3 | 1996 |
| 6 | limit 5 to english language | 1253 |
| 7 | limit 6 to yr=”1990 -Current” | 650 |

Ovid Embase 30/9/16

| **Search #** | **Search term** | **Results** |
| --- | --- | --- |
| 1 | cauda equina syndrome.af. | 2580 |
| 2 | (case report or abstract).af. | 13016786 |
| 3 | animal.af. | 5369655 |
| 4 | (cauda equina syndrome not (case report or abstract)).af. | 1191 |
| 5 | (cauda equina syndrome not (case report or abstract) not animal).af. | 1116 |
| 6 | limit 5 to english language [Limit not valid in Your Journals@Ovid; records were retained] | 993 |
| 8 | Limit 9 to yr=”1990 –Current” | 949 |

CINAHL Plus 30/9/16

| **Search #** | **Search term** | **Search Options** | **Results** |
| --- | --- | --- | --- |
| 1 | Cauda equina syndrome |  | 330 |
| 2 | Cauda equina syndrome NOT (case report or abstract) |  | 252 |
| 3 | Cauda equina syndrome NOT (case report or abstract) NOT (animal) |  | 246 |
| 4 | Cauda equina syndrome NOT (case report or abstract) NOT (animal) | **Narrow by Language:**- english | 241 |
| 5 | Cauda equina syndrome NOT (case report or abstract) NOT (animal) | **Narrow by Language:**- english  **Limiters** - Publication Year: 1990-2016 | 239 |
